# Targets of Vitamin C With Therapeutic Potential for Cardiovascular Disease and Underlying Mechanisms: A Study of Network Pharmacology

**DOI:** 10.3389/fphar.2020.591337

**Published:** 2021-02-02

**Authors:** Ning Zhu, Bingwu Huang, Wenbing Jiang

**Affiliations:** ^1^ Department of Cardiology, The Third Affiliated Hospital of Shanghai University, The Wenzhou Third Clinical Institute Affiliated to Wenzhou Medical University, Wenzhou People’s Hospital, Wenzhou, China; ^2^ Department of Anesthesiology, The Third Affiliated Hospital of Shanghai University, The Wenzhou Third Clinical Institute Affiliated to Wenzhou Medical University, Wenzhou People’s Hospital, Wenzhou, China

**Keywords:** vitamin C, cardiovascular disease, network pharmacology, inflammation, jak-stat

## Abstract

Vitamin C (ascorbic acid) is a nutrient used to treat cardiovascular disease (CVD). However, the pharmacological targets of vitamin C and the mechanisms underlying the therapeutic effects of vitamin C on CVD remain to be elucidated. In this study, we used network pharmacology approach to investigate the pharmacological mechanisms of vitamin C for the treatment of CVD. The core targets, major hubs, enriched biological processes, and key signaling pathways were identified. A protein-protein interaction network and an interaction diagram of core target-related pathways were constructed. Three core targets were identified, including phosphatidylinositol 4,5-bisphosphate 3-kinase catalytic subunit alpha isoform, signal transducer and activator of transcription-3 (STAT3), and prothrombin. The GO and KEGG analyses identified top 20 enriched biological processes and signaling pathways involved in the therapeutic effects of vitamin C on CVD. The JAK-STAT, STAT, PD1, EGFR, FoxO, and chemokines signaling pathways may be highly involved in the protective effects of vitamin C against CVD. In conclusion, our bioinformatics analyses provided evidence on the possible therapeutic mechanisms of vitamin C in CVD treatment, which may contribute to the development of novel drugs for CVD.

## Introduction

Cardiovascular disease (CVD) is a leading cause of death worldwide, accounting for 205 deaths per 100,000 persons (Yu et al., 2019). It represents a substantial proportion of healthcare spending, therefore placing an enormous financial burden on patients and their families (Evans et al., 2020). CVD is characterized by a cluster of disorders in the arteries and heart that result in atherosclerosis, hypertension, myocardiopathy, myocardial infarction, and heart failure (North and Sinclair, 2012). The mechanism underlying the pathogenesis of CVD is complex and has not been fully elucidated (Buckley et al., 2019). To develop better treatment for patients with CVD, studies have been launched to identify novel therapeutic targets for the past two decades (Touzé and Rothwell, 2007; O’Donnell and Nabel, 2011; Khera and Kathiresan, 2017). Drugs targeting beta receptor, RASS system, P2Y12 receptor, PCSK9, and HMG CoA reductase have been shown to decrease cardiovascular morbidity and mortality in CVD patients (Ference et al., 2017; Squizzato et al., 2017; Blessberger et al., 2018). Despite great advances in treatment, CVD remains the dominant cause of mortality worldwide (Feng et al., 2019). Hence, there is an urgent need to develop novel therapeutic strategies and drugs for the treatment of CVD.

Vitamin C (ascorbic acid) is a nutrient with radical scavenger activity and antioxidant effects (May and Harrison, 2013). Observational studies have demonstrated that high vitamin C supplementation is inversely correlated with the risk of CVD (Salonen et al., 2003; Wang et al., 2013). A systematic meta-analysis of 44 randomized controlled trials (RCTs) suggested that vitamin C intake improved left ventricular ejection fraction in patients with heart failure (Ashor et al., 2014). It has also been shown that vitamin C intake is associated with low blood pressure in hypertensive participants (Juraschek et al., 2012; Ran et al., 2020). In addition, vitamin C supplementation decreased cardiovascular mortality in a cohort of Spanish graduates (Martín-Calvo and Martínez-González, 2017).

The antioxidant property of vitamin C contributes to the prevention and treatment of cardiovascular disorders (Ingles et al., 2020). However, the molecular mechanisms remain elusive. Few studies have investigated the signaling pathways involved in the effects of vitamin C on CVD. The network pharmacology-based approach has been used to identify novel therapeutic targets and illustrate the molecular mechanisms of vitamin C against sepsis (Li et al., 2020a). We also elucidated the pharmacological mechanisms of the active ingredients of traditional Chinese medicines on the treatment of CVD (Huang et al., 2020).

In the present study, we investigated the molecular mechanisms underlying the protective effects of vitamin C against CVD by using network pharmacology approach. Our bioinformatics data may contribute to the development of new treatments for CVD for both clinical and basic investigators.

## Methods

### Prediction of Putative Targets of Vitamin C With Therapeutic Potential for Cardiovascular Disease

Swiss Target Prediction (http://www.swisstargetprediction.ch), DrugBank (http://www.drugbank.ca/) database, and Traditional Chinese Medicine Systems Pharmacology (TCMSP, http://lsp.nwu.edu.cn/tcmsp.php) were used to obtain all known targets of vitamin C. The therapeutic targets for CVD were collected using DrugBank database, Online Mendelian Inheritance (OMIM) (http://www.omim.org/) database, and GeneCards (www.genecards.org/) database. Then the targets of vitamin C with therapeutic potential for CVD were identified after eliminating duplicates.

### Construction of Protein-Protein Interaction Network and Topological Analysis of Vitamin C Against Cardiovascular Disease

STRING database was used to construct a target-to-target, function-related, protein-protein interaction (PPI) network and to obtain general data (tsv.). Protein interactions with a confidence score of >0.9 were selected. General data were imported into Cytoscape (v3.7.1) to rebuild a PPI network of vitamin C against CVD. The network analyzer in Cytoscape was used to analyze the topological parameters, including mean and maximum degrees of freedom in the PPI network. The upper limit of the screening range was the maximum degree value in the topological data, while the lower limit was twice the average degree of freedom (Su et al., 2019). The core targets were identified based on the above settings.

### Functional Processes and Pathway Analysis

The R packages of ClusterProfiler, enrichplot, and ggplot2 were used for Gene Ontology (GO) Biological Function (BP) and Kyoto Encyclopedia of Genes and Genomes (KEGG) pathway enrichment analyses of the core targets. The cut-off value was set as *p* < 0.05.

### Construction of Network Relationships

**FIGURE 1:**
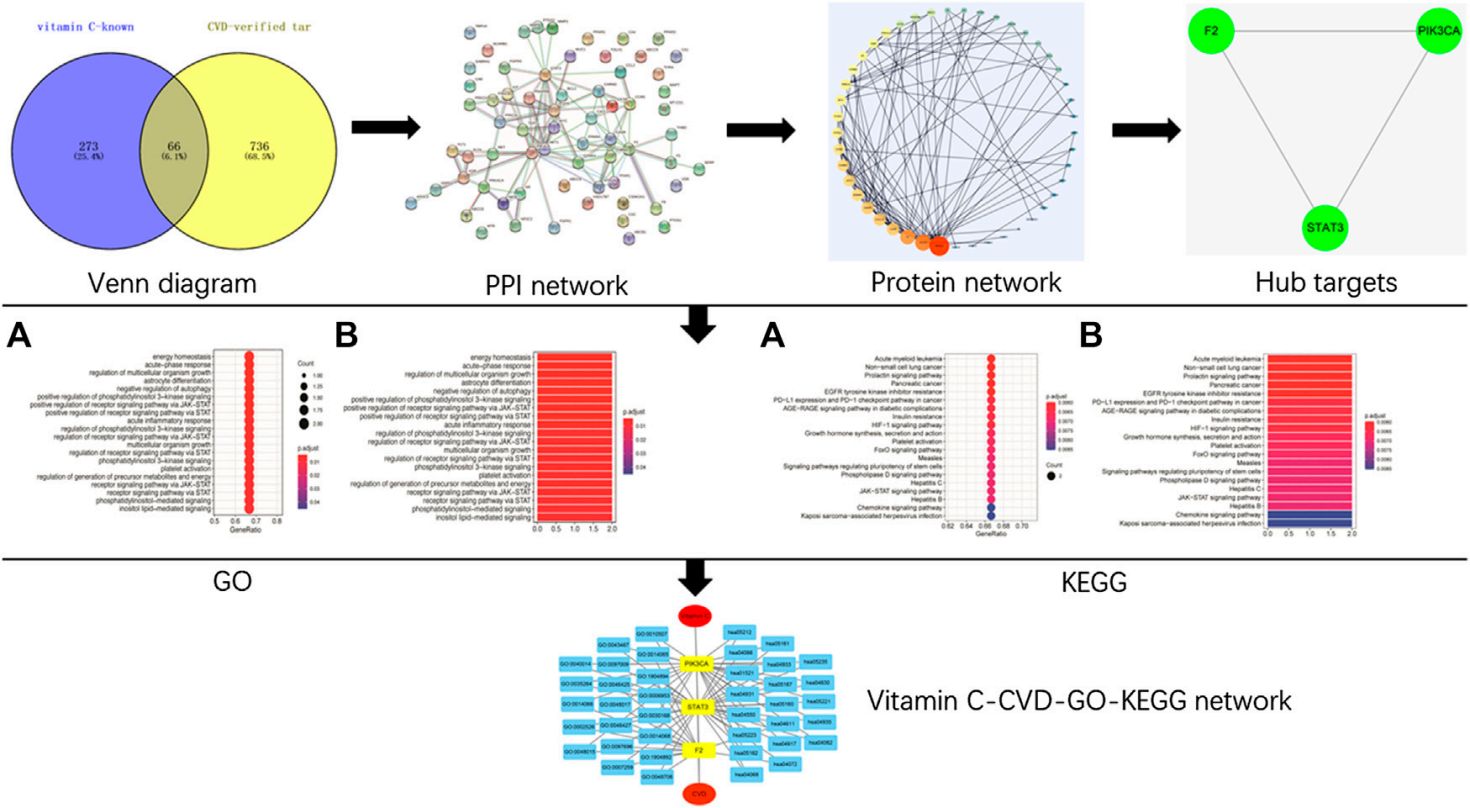
A schematic diagram of network pharmacology approach for the identification of core targets, major hubs, PPI network, biological processes, and key pathways of vitamin C acting on CVD. All known targets of vitamin C and CVD were predicted using online databases. Then the targets of vitamin C with therapeutic potential for CVD were identified. After the construction of a PPI network and the identification of the hub targets of vitamin C acting on CVD, GO BP, and KEGG pathway enrichment analyses were performed. Finally, a network of vitamin C-CVD -GO-KEGG was generated.

Cytoscape (v3.7.1) software was used for network visualization of the targets of vitamin C against CVD. An interaction diagram of core target-related GO/KEGG enrichment was generated (Wu et al., 2019). The study design is shown in Figure 1.

## Results

### Known Targets of Vitamin C With Therapeutic Potential for Cardiovascular Disease

**FIGURE 2:**
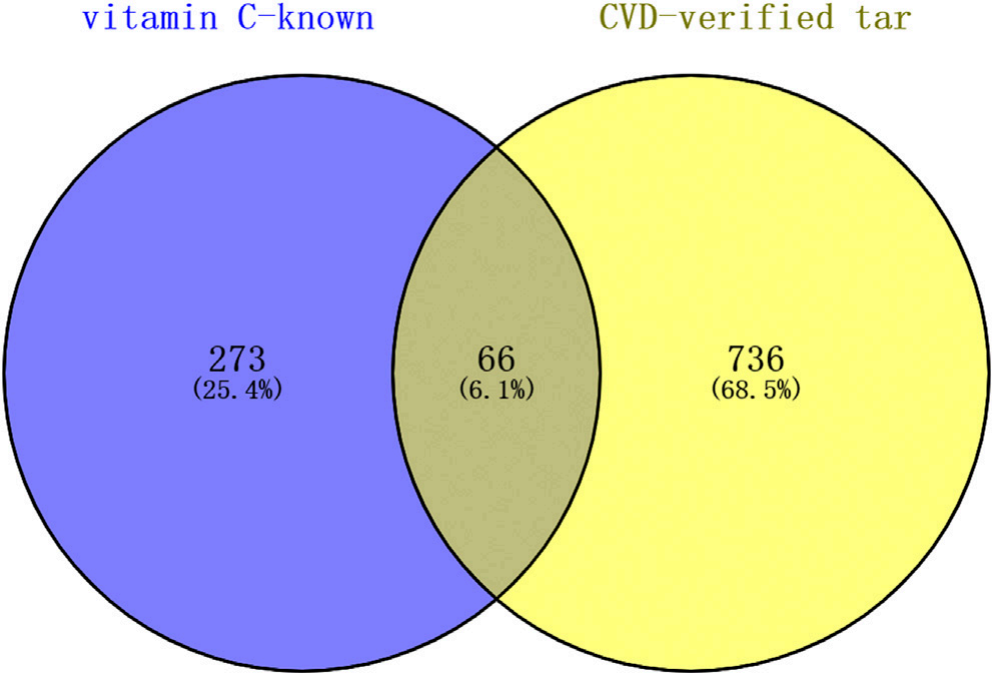
The targets of vitamin C with therapeutic potential for CVD were identified using the Venn diagram. Swiss Target Prediction, DrugBank database, and TCMSP were used to obtain all known targets of vitamin C. DrugBank database, OMIM database, and GeneCards database were used to collect all therapeutic targets for CVD. A total of 66 targets of vitamin C with therapeutic potential for CVD were identified.

**FIGURE 3:**
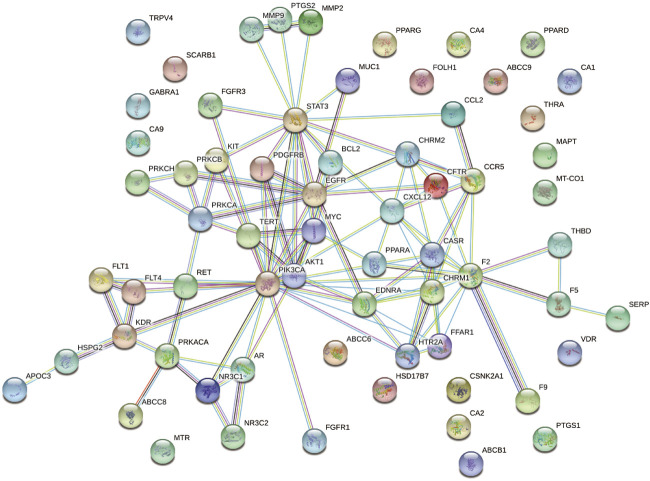
A PPI network of the targets of vitamin C with therapeutic potential for CVD was generated. A PPI network of 66 targets was imported into STRING database and visualized with the interaction score set to the highest confidence (0.900).

A total of 339 known target genes of vitamin C and 802 known therapeutic targets for CVD were obtained (Supplementary Tables S1, S2). The Venn diagram was plotted using online accessible tools to identify the targets of vitamin C against CVD (Figure 2). Finally, 66 targets of vitamin C with therapeutic potential for CVD were identified and then analyzed using STRING database. The function-related PPI network is shown in Figure 3.

### Topology Parameter Analysis and Identification of Core Targets

**FIGURE 4:**
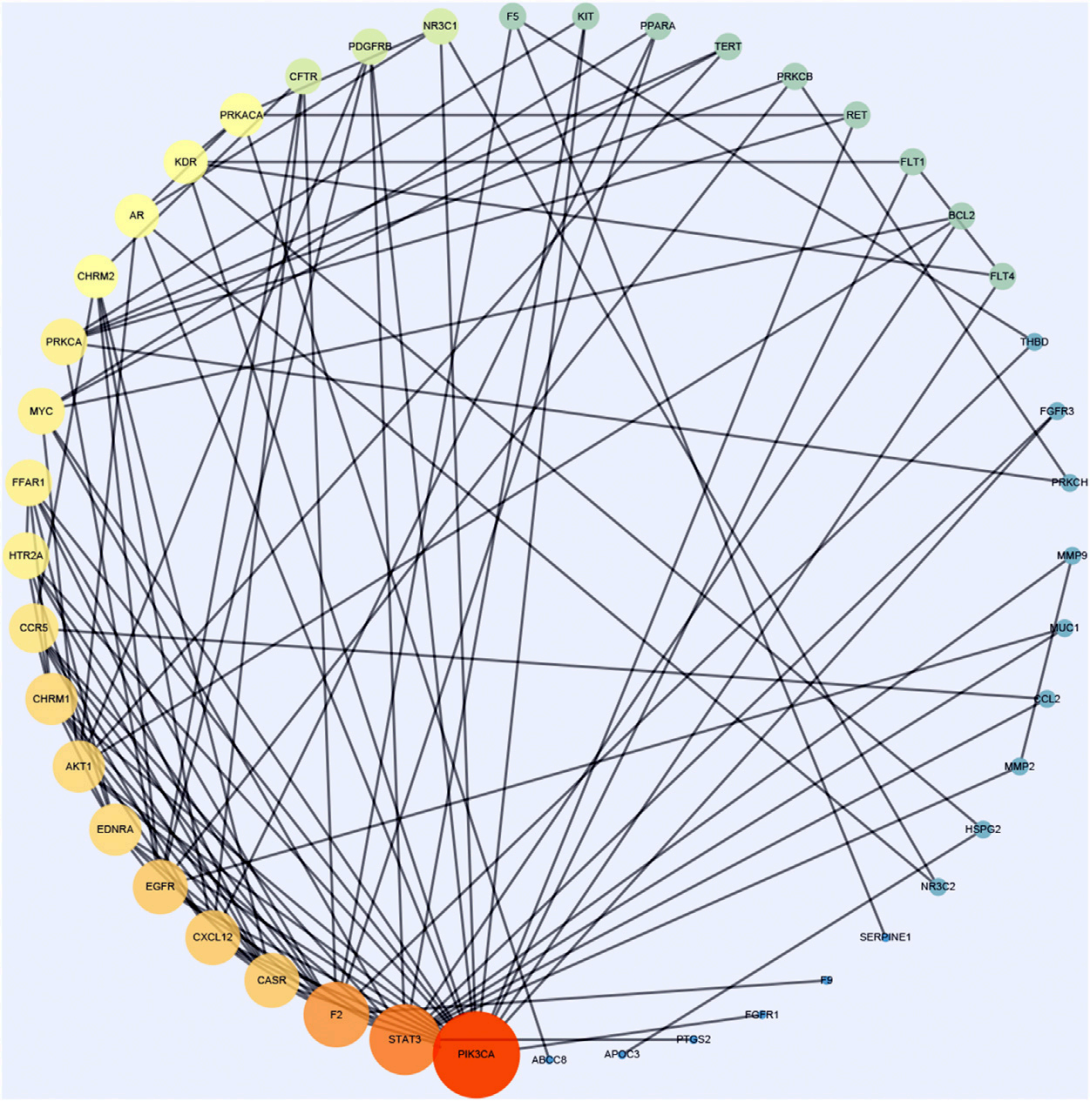
A protein network of known targets of vitamin C with therapeutic potential for CVD was generated by Cytoscape. The targets were imported into Cytoscape for the analysis of topological parameters. The diagram was generated based on topological analysis.

**FIGURE 5:**
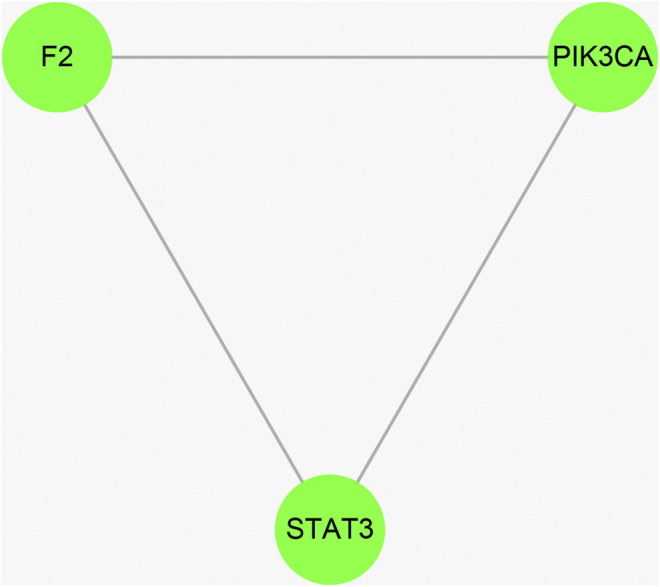
The hub targets of vitamin C with therapeutic potential for CVD were identified using the Cytoscape software. The median and maximum degrees of freedom of the target were 1 and 21, respectively. To screen the hub targets, the degree of freedom was set to >12.

Cytoscape was used to calculate the topological parameters of the interaction network of 66 identified targets (Figure 4). The minimum degree of freedom of the target was one and the maximum degree of freedom was 21. The screening criteria were set to 12 and 21 for the core targets. A total of three core targets were determined, including phosphatidylinositol 4,5-bisphosphate 3-kinase catalytic subunit alpha isoform (PIK3CA), signal transducer and activator of transcription-3 (STAT3), and prothrombin (F2) (Figure 5).

### Gene Ontology Biological Function and Kyoto Encyclopedia of Genes and Genomes Pathway Enrichment Analysis

**FIGURE 6:**
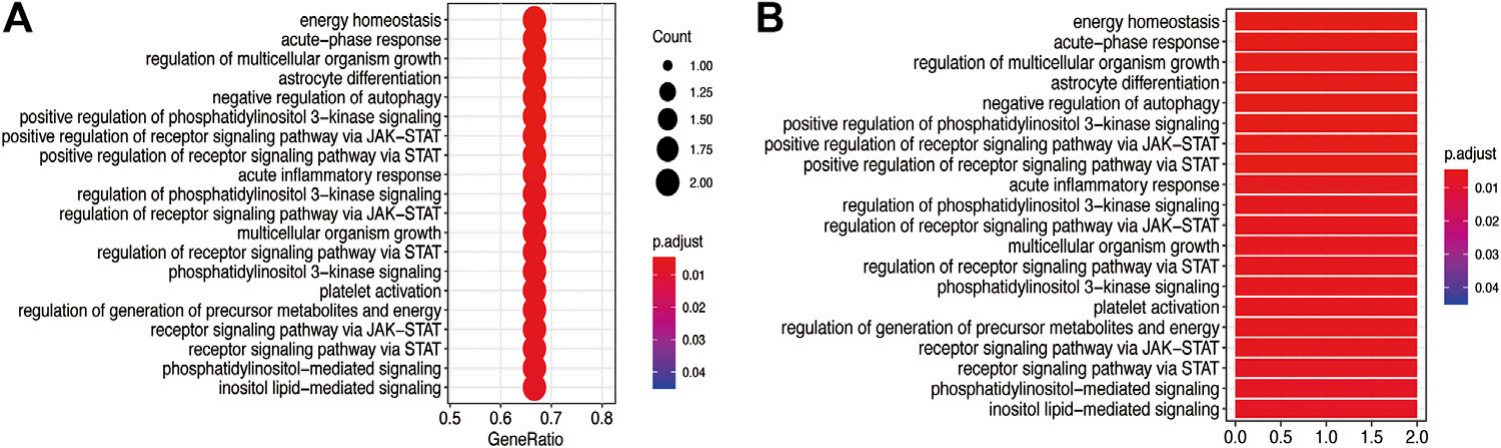
GO BP analysis of the core targets. GO BP enrichment analysis of the three core targets was performed using R language. **(A)** The bubble diagram shows the top 20 enriched BP. The *x*-axis represents the gene ratio and the intensities of different colors indicate the adjusted *p*-value. **(B)** The histogram shows the top 20 enriched BP. The *x*-axis represents the enriched gene count and the intensities of different colors indicate the adjusted *p*-value.

**FIGURE 7:**
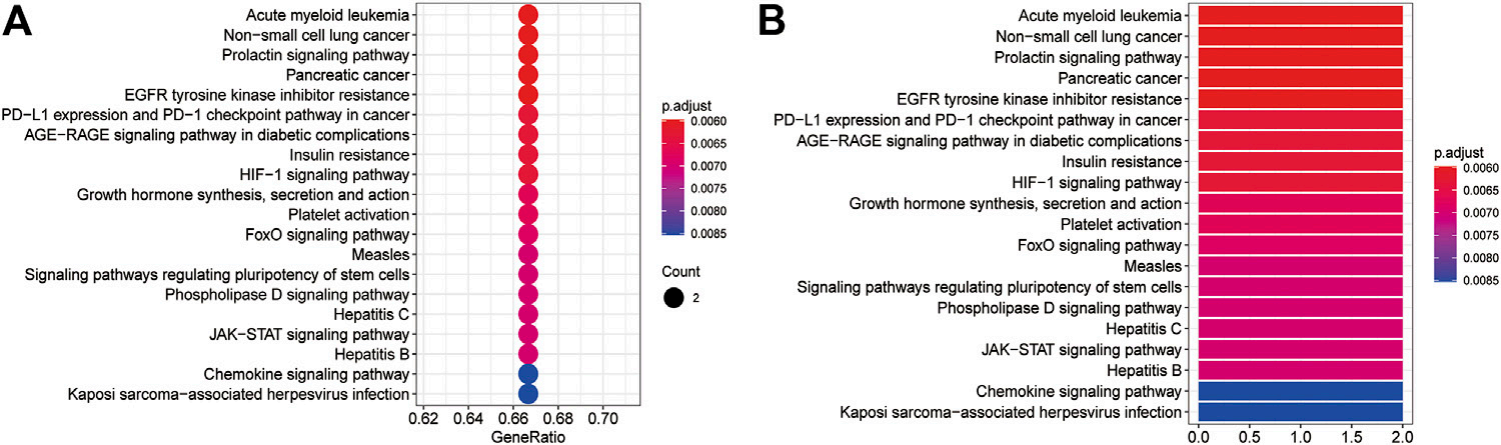
The KEGG pathway enrichment analysis of the core targets. The KEGG pathway enrichment analysis was performed using R language. **(A)** The bubble diagram shows the top 20 enriched KEGG pathways. The *x*-axis represents the gene ratio and the intensities of different colors indicate the adjusted *p*-value. **(B)** The histogram shows the top 20 enriched KEGG pathways. The *x*-axis represents the enriched gene count and the intensities of different colors indicate the adjusted *p*-value.

The GO BP and KEGG pathway enrichment analyses of three core targets were performed using R language. The enriched biological functions included regulation of energy homeostasis, acute-phase response, regulation of multicellular organism growth, regulation of multicellular organism growth, astrocyte differentiation, negative regulation of autophagy, positive regulation of phosphatidylinositol 3-kinase signaling, positive regulation of receptor signaling pathway via JAK-STAT, positive regulation of receptor signaling pathway via STAT, acute inflammatory response, regulation of phosphatidylinositol 3-kinase signaling, regulation of receptor signaling pathway via JAK-STAT, multicellular organism growth, regulation of receptor signaling pathway via STAT, phosphatidylinositol 3-kinase signaling, platelet activation, regulation of generation of precursor metabolites and energy, receptor signaling pathway via JAK-STAT, receptor signaling pathway via STAT, phosphatidylinositol-mediated signaling, and inositol lipid-mediated signaling (Figure 6). The enriched molecular signaling pathways of the core targets were involved in acute myeloid leukemia, non-small cell lung cancer, prolactin signaling pathway, pancreatic cancer, EGFR tyrosine kinase inhibitor resistance, PD-L1 expression and PD-1 checkpoint pathway in cancer, AGE-RAGE signaling pathway in diabetic complications, insulin resistance, HIF-1 signaling pathway, growth hormone synthesis, secretion and action, platelet activation, FoxO signaling pathway, measles, signaling pathways regulating pluripotency of stem cells, phospholipase D signaling pathway, Hepatitis C, JAK-STAT signaling pathway, Hepatitis B, chemokines signaling pathway, and Kaposi’s sarcoma-associated herpesvirus infection (Figure 7).

### Network Construction

**FIGURE 8:**
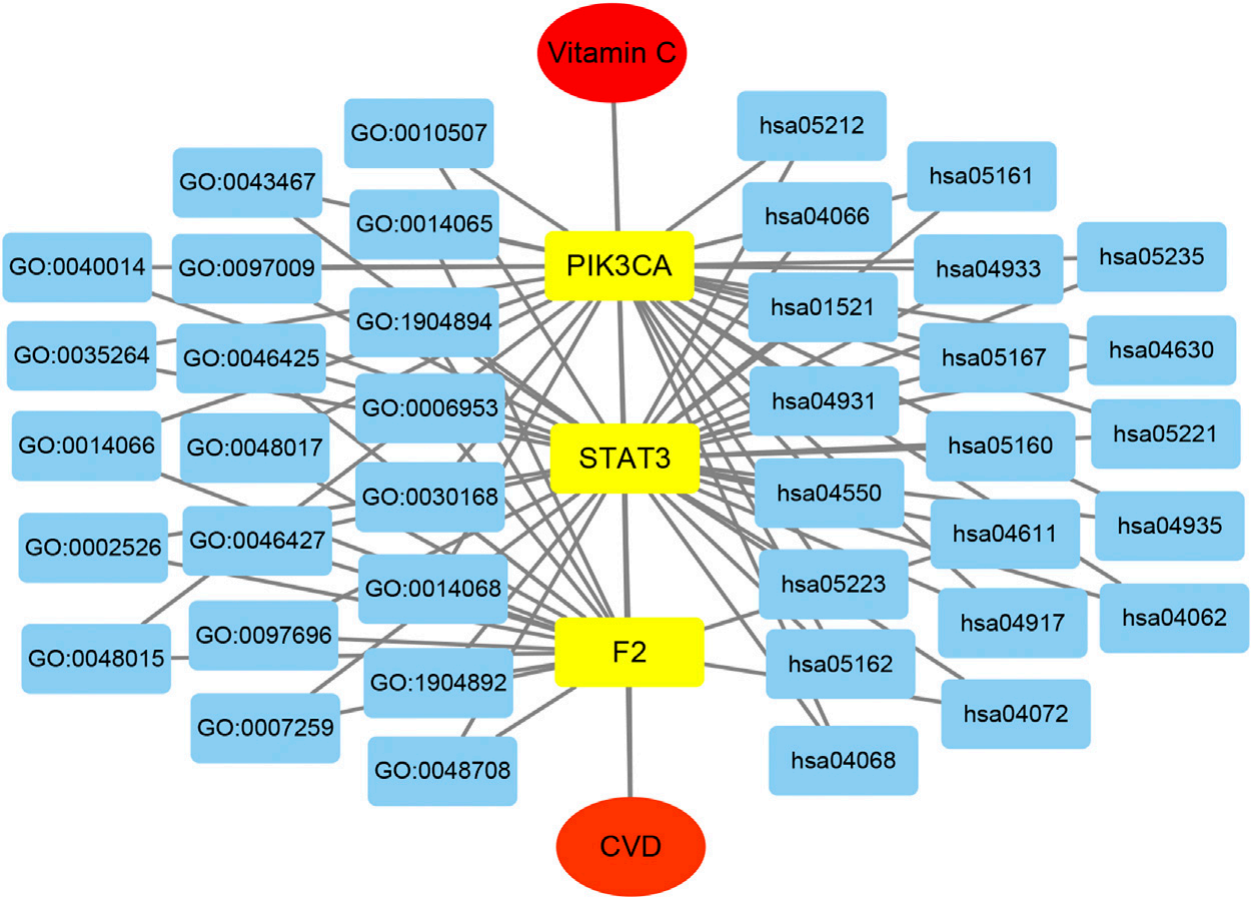
The integrated vitamin C-CVD-GO-KEGG network was visualized by Cytoscape. The yellow rectangles represent three core targets. The blue rectangles show the top 20 enriched GO BP and KEGG pathways of vitamin C acting on CVD.

The network of the core targets of vitamin C with therapeutic potential for CVD and the interaction diagram of the core target-related pathways were constructed (Figure 8).

## Discussion

Oxidative stress and inflammation play a crucial role in the onset and progression of atherosclerosis and the development of cardiovascular events (Pignatelli et al., 2018; Ilatovskaya et al., 2019). Inflammation promotes oxidative stress, which in turn leads to further inflammation, therefore establishing a self-perpetuating cycle. Methotrexate and doxycycline, two medications with antioxidant properties, exhibit therapeutic benefits in patients with CVD, possibly by inhibiting inflammation and breaking the self-perpetuating cycle (Clemens et al., 2020). Numerous studies have shown that reduced inflammation attenuated CVD. Statins reduce both LDL cholesterol and high-sensitivity C-reactive protein (hs-CRP), a biomarker of inflammation (Ridker et al., 1999; Albert et al., 2001). Moreover, a clinical trial showed that treatment with canakinumab, a monoclonal antibody targeting interleukin (IL)-1β, significantly downregulated hs-CRP and reduced the occurrence of cardiovascular events (Harrington 2017; Ridker et al., 2017). However, treatment efficacy of other inflammation inhibitors for CVD needs to be evaluated in future clinical studies. The role of oxidative stress and inflammation in CVD remain to be deeply and systematically investigated. The identification of new therapeutic targets and the elucidation of novel regulatory mechanisms of existing drugs enable the development of treatments for CVD. The network pharmacology approach is an innovative method to systematically explore the mechanisms of the effects of drugs on disease. It consists of bioinformatics, network analysis and integrates multiple sources of information. Hence, network approaches can accurately determine potential interactions between drug and target, and promote drug discovery. In this study, we used network pharmacology to identify the core targets of vitamin C with therapeutic potential for CVD and performed GO BP and KEGG enrichment analyses. Bioinformatics findings can reveal the proposed pharmacological mechanism and strengthen the new ideas for CVD treatment (Zhang et al., 2020).

Previous studies have extensively reported the beneficial effects of vitamin C on CVD. However, large RCTs did not identify any benefit of vitamin C (and E) intake on CVD (Cook et al., 2007; Sesso et al., 2008). Furthermore, a meta-analysis of RCTs showed that vitamin C supplementation has no effects on major cardiovascular outcomes such as CVD mortality and myocardial infarction (Al-Khudairy et al., 2017). There is still a need to identify the role of vitamin C in mitigating cancer treatment-induced heart failure (Malik et al., 2020) and other risks of CVD, including cardiomyopathies, arrhythmias and myocarditis/pericarditis in long-term cancer survivors (Berretta et al., 2020). However, the evidence is limited because many trials of vitamin C (and E) supplementation did not report protective effects on risks of CVD, such as blood pressure, arterial stiffness, endothelial function, or left ventricular ejection fraction. These risks of CVD may be more sensitive to vitamin C (and E) supplementation because they are responsible for the damaging effects of inflammation and oxidative stress (Pashkow, 2011). Moreover, they occur in the early stage of CVD pathogenesis. The potential protective effects of vitamin C intake on CVD remains controversial in both preclinical and clinical studies (Morelli et al., 2020). Therefore, to promote novel drug discovery, the mechanisms underlying the therapeutic effects of vitamin C on CVD should be well determined. Here, three core targets of vitamin C against CVD were identified: PIK3CA, STAT3, and F2. The GO analysis revealed that the most enriched BP was positive regulation of the STAT signaling pathways, which may play a critical role in CVD. The JAK-STAT pathway, which can be activated by a range of cytokines (i.e., IL-6, IL-2, and interferons), regulate the survival, proliferation, and differentiation of various types of cells (Speirs et al., 2018). In addition, the excessive activation of the JAK-STAT signaling is a key driver of many chronic inflammatory diseases, including CVD (Jones et al., 2011; Sansone and Bromberg, 2012). The four JAKs (JAK1-3 and Tyk2) comprise a family of cytoplasmic tyrosine kinases. Inflammatory cytokines, such as IL-6, promote the activation of JAKs, leading to subsequent phosphorylation of gp130 on Tyr residues, which generates docking sites for SH2-domain-containing STAT proteins (Schaper and Rose-John, 2015). JAKs induce Tyr-phosphorylation of STATs within their SH2 domains (Tyr701 on STAT1, Tyr705 on STAT3). The activated STATs translocate to the nucleus to drive the transcription of inflammatory target genes (Mao et al., 2005; O’Shea et al., 2013). Vitamin C is a potent anti-inflammatory drug (Williams et al., 2019) that has been used to treat inflammatory diseases, such as obesity, sepsis, and pneumonia (Gorton and Jarvis, 1999; Ellulu., 2017; Marik, 2018). In the present study, we showed the therapeutic effects of vitamin C on CVD were associated with its anti-inflammatory property, more specifically, the inhibition of the JAK-STAT signaling. Therefore, drugs targeting the JAK-STAT signaling may be developed for the treatment of CVD.

In agreement with GO analysis, KEGG pathway enrichment analysis also showed that the therapeutic effects of vitamin C on CVD were mainly associated with the JAK-STAT signaling pathway, as well as the PD-1 pathway, EGFR tyrosine kinase inhibitor resistance, the FoxO signaling pathway, and the chemokines signaling pathway (Li et al., 2020a; Li et al., 2020b). There is crosstalk among STAT, PD-1, EGFR, and FoxO signals. It was reported that the upregulation of EGFR promoted COPD airway epithelial cells by regulating the FOXO signaling pathway (Ganesan et al., 2013). JAK-STAT and EGFR together specify a population of cells called the posterior follicle cells to establish the embryonic axes (Wittes and Schüpbach, 2019). The expression of PD-L1 was induced by EGFR and JAK2/STAT1, while the inhibition of JAK2 repressed the upregulation of PD-L1 in tumor cells and enhanced their immunogenicity (Concha-Benavente et al., 2016). A clinical study showed that patients with EGFR mutation had increased PD-L1 expression and T cell infiltration (Chen et al., 2020). The activation of STAT resulted in upregulated PD-L1 expression and the progression of lymphoma (Estrada et al., 2018). The activation of EGFR was implicated in CVD via the regulation of blood pressure, endothelial dysfunction, neointimal hyperplasia, atherogenesis, and cardiac remodeling (Makki et al., 2013). In addition, FOXOs were identified as therapeutic targets in several major cardiac diseases, such as ischemic cardiac diseases, diabetic cardiomyopathy, and myocardial hypertrophy (Xin et al., 2017). The expression level of PD-1 can affect the degree of inflammation and the state of coronary plaques in atherosclerosis (Sun et al., 2020). The crosstalk among these signaling pathways result in excessive inflammation of heart and vessels. In this study, we demonstrated that these signaling pathways play crucial roles in the progression of CVD. The network pharmacology provided a better understanding of the role of inflammation in CVD at systematic level. Further studies are needed to determine the role of these pathways in CVD and the mechanisms involved in the regulatory processes.

KEGG pathway enrichment showed that the chemokines signaling pathway was implicated in the therapeutic effects of vitamin C against CVD. The secreted chemokines cause epidermal damage by attracting proinflammatory immunocytes (Bellón, 2019). The role of immunocytes in CVD has been studied. Neutrophils promote atherosclerosis at different stages, including atherogenesis, plaque destabilization, and plaque erosion (Silvestre-Roig et al., 2020). They are also involved in the pathogenic and repair processes in heart failure, myocardial infarction, and neointima formation. A number of experimental and clinical studies have indicated that T cells protect against cardiovascular disease, particularly atherosclerosis and abdominal aortic aneurysm (Meng et al., 2016). Other clinical researchers suggested that therapeutic strategies targeting B cells might exhibit beneficial effects on CVD (Porsch and Binder, 2019). Our data indicated that the blockage of the chemokines signaling pathway and immune response could reduce the progression of CVD. Because of the crosstalk among inflammation, oxidative stress, immune response, and chemokines, combination drug treatment may be a more useful approach for the treatment of CVD.

## Conclusion

In summary, we identified three core targets (PIK3CA, STAT3, and F2) of vitamin C with therapeutic potential for CVD and showed that the protective effects of vitamin C on CVD were attributed to its anti-inflammation property. The most enriched pathways were the JAK-STAT, PD-1, EGFR, FoxO, and chemokines signaling pathways. Our findings may guide further pharmacological investigations on the therapeutic effects of vitamin C on CVD and the discovery of new drugs for CVD treatment.

### Limitation

There are some limitations to the current study. First, the targets of vitamin C with therapeutic potential for CVD were collected using public databases, which may lead to inaccurate results. Second, the core targets identified in the current study need to be further validated. Third, as CVD is a complex process involving different types of diseases, the roles of these targets and pathways in specific pathological conditions need to be evaluated in both basic scientific and clinical studies.

## Data Availability Statement

The original contributions presented in the study are included in the article/Supplementary Material, further inquiries can be directed to the corresponding author.

## Author Contributions

NZ: designed, drafted, and revised the manuscript. BWH: performed the research and wrote the manuscript. WBJ: collected and analyzed the data. All the authors read and approved the final manuscript.

## Funding

This research was supported by Natural Science Foundation of Zhejiang Province (No. LQ20H020011).

## Conflict of Interest

The authors declare that the research was conducted in the absence of any commercial or financial relationships that could be construed as a potential conflict of interest.
